# The effect of xanthine oxidase and hypoxanthine on the permeability of red cells from patients with sickle cell anemia

**DOI:** 10.14814/phy2.13626

**Published:** 2018-03-04

**Authors:** Halima W. M. Al Balushi, David C. Rees, John N. Brewin, Anke Hannemann, John S. Gibson

**Affiliations:** ^1^ Department of Veterinary Medicine University of Cambridge Cambridge United Kingdom; ^2^ Department of Paediatric Haematology King's College Hospital King's College London London United Kingdom

**Keywords:** Oxidants, red cell permeability, sickle cell anemia, xanthine oxidase

## Abstract

Red cells from patients with sickle cell anemia (SCA) are under greater oxidative challenge than those from normal individuals. We postulated that oxidants generated by xanthine oxidase (XO) and hypoxanthine (HO) contribute to the pathogenesis of SCA through altering solute permeability. Sickling, activities of the main red cell dehydration pathways (P_sickle_, Gardos channel, and KCl cotransporter [KCC]), and cell volume were measured at 100, 30, and 0 mmHg O_2_, together with deoxygenation‐induced nonelectrolyte hemolysis. Unexpectedly, XO/HO mixtures had mainly inhibitory effects on sickling, P_sickle_, and Gardos channel activities, while KCC activity and nonelectrolyte hemolysis were increased. Gardos channel activity was significantly elevated in red cells pharmacologically loaded with Ca^2+^ using the ionophore A23187, consistent with an effect on the transport system *per se* as well as via Ca^2+^ entry likely via the P_sickle_ pathway. KCC activity is controlled by several pairs of conjugate protein kinases and phosphatases. Its activity, however, was also stimulated by XO/HO mixtures in red cells pretreated with *N*‐ethylmaleimide (NEM), which is thought to prevent regulation via changes in protein phosphorylation, suggesting that the oxidants formed could also have direct effects on this transporter. In the presence of XO/HO, red cell volume was better maintained in deoxygenated red cells. Overall, the most notable effect of XO/HO mixtures was an increase in red cell fragility. These findings increase our understanding of the effects of oxidative challenge in SCA patients and are relevant to the behavior of red cells in vivo.

## Introduction

Sickle cell anemia (SCA) is one of the commonest severe inherited disorders affecting millions of people worldwide (Piel et al. 2013). Etiology is caused by inheritance of the mutated hemoglobin, HbS, which has a single amino acid substitution when compared with normal adult Hb, HbA, with valine replacing glutamic acid at the sixth residue of the two *β* chains of the Hb tetramer (Bunn and Forget 1986). The loss of a negative charge at this crucial position on the surface of the HbS protein allows it to polymerize upon deoxygenation forming long, rigid rods. The ensuing sickling shape change, adverse rheology, and other harmful sequelae underlie the multiple clinical signs of SCA. Although details of the pathogenesis remain unclear, vascular occlusion is a key event. Complications include pain, acute chest syndrome, stroke, nephropathy, osteonecrosis, leg ulcers, and reduced lifespan, although both the frequency and severity of these problems vary markedly between patients, (Steinberg 1999; Rees et al. 2010).

Mainstream treatment largely revolves around three management strategies – transfusion to dilute sickling red cells, antibiotic therapy or vaccination to tackle pneumococcal and other infections, and nonspecific measures to provide support to the organ(s) most affected (Rees et al. 2010). Dating from the 1980s, hydroxyurea has emerged as the only specific reagent licensed to treat SCA patients (Platt et al. 1984; Charache et al. 1987). It probably works mainly by increasing the expression of HbF levels which reduces the interaction between HbS molecules, thereby limiting its tendency to polymerize. Hydroxyurea is not without problems, however, and although its use is increasing, it remains largely confined to individuals with significant symptoms (Rees 2011). Other fruitful approaches have included the design of compounds which increase the oxygen affinity of HbS, to promote the oxy conformation of HbS and thereby inhibit polymerization. Many such reagents are derivatives of aromatic aldehydes and have included vanillin, 5‐hydroxymethylfurfural (5HMF, Aes103) and more recently GBT440 (Abraham et al. 1991; Abdulmalik et al. 2005; Oksenberg et al. 2016), but, to date, none has progressed to clinical use. A better understanding of pathogenesis would enable rational design of novel and more effective treatments.

A key feature of SCA of pathogenic importance is increased oxidative stress within the vasculature and in which red cells, whose close relationship with oxygen during its transport from lungs to tissues, represent an obvious target (Hebbel et al. 1982; Rice‐Evans et al. 1986; Aslan et al. 2000; Chirico and Pialoux 2012; Voskou et al. 2015). Oxidative challenge may occur either endogenously within the red cells themselves or exogenously coming from other tissues. In addition, the normal protective antioxidant capacity of red cells is often thought to be reduced in SCA patients (Gizi et al. 2011; Silva et al. 2013).

Within HbS‐containing red cells, increased levels of reactive oxygen species (ROS) are generated by the relative instability of HbS compared with normal HbA. Autoxidation of Hb and ROS production by the Fenton reaction occur faster than for HbA‐containing red cells with accumulation of heme, hemichromes, and iron, and subsequent generation of ROS (Hebbel et al. 1982, 1988; Rice‐Evans et al. 1986; Banerjee and Kuypers 2004). The absolute oxygen tension is significant, as partially deoxygenated Hb shows a marked increase in the rate of autoxidation (Abugo and Rifkind 1994; Balagopalakrishna et al. 1996; Mohanty et al. 2014). Recently, red cell NADPH oxidases have also been shown to generate intracellular oxidants, and have a higher activity in cells from SCA patients, perhaps following stimulation by circulating proinflammatory cytokines (George et al. 2013). Exogenously, ROS arise from repeated episodes of ischemia and reperfusion, which are frequent occurrences in vaso‐occlusive disorders like SCA (Zweier and Talukder 2006). Xanthine oxidase (XO) may be released from damaged tissues (Balagopalakrishna et al. 1996; Lard et al. 1999; Aslan et al. 2001; Wun 2001; Voskou et al. 2015). It produces varying amounts of hydrogen peroxide and superoxide anion (Kelley et al. 2010), both of which can gain access to the red cell cytoplasm via the anion exchanger (or band 3) (Rogers et al. 2009; Voskou et al. 2015) or damage membrane lipid and/or protein components. Intracellularly, several reactions metabolize the oxidants produced including red cell Zn^2+^/Cu^2+^ superoxide dismutase which together with heme iron forms various other ROS such as hydroxyl radicals. ROS production is highest when Hb is about 60% saturated with oxygen (Balagopalakrishna et al. 1996). ROS are also formed in activated white cells and vascular endothelium (Lard et al. 1999; Aslan et al. 2001; Wun 2001; Voskou et al. 2015).

A further complication in red cells from SCA patients is their increased solute permeability. This is significant because it causes red cells to lose KCl, with water following osmotically. The reduction in volume serves to increase the intracellular concentration of HbS, which is critically important in pathogenesis as it markedly encourages HbS polymerization by reducing the lag time for polymer formation following deoxygenation (Eaton and Hofrichter 1987). Three transport systems are intimately involved in solute loss (Joiner 1993; Gibson 2001; Lew and Bookchin 2005): a deoxygenation‐induced cation conductance termed P_sickle_, the Ca^2+^‐activated K^+^ channel (or Gardos channel), and the KCl cotransporter. Activities of all three transport systems are enhanced, uniquely present, or abnormally regulated in red cells from SCA patients. P_sickle_ may mediate Ca^2+^ entry to stimulate the Gardos channel and also promote Ca^2+^‐dependent phospholipids scrambling leading, in addition, to phosphatidylserine (PS) exposure with increased stickiness and prothrombotic activity (Zwaal and Schroit 1997) of sickle red cells. It also causes Mg^2+^ depletion, which may further stimulate KCl cotransporter (KCC) activity (Delpire and Lauf 1991).

A number of oxidants including nitrite (Muzyamba et al. 2000), phenazine methosulfate (Gibson and Muzyamba 2003a,b), 1‐chloro‐2,4‐dinitrobenzene (Gibson and Muzyamba 2003a,b), and peroxynitrite (Kucherenko et al. 2005) have previously been found to have significant stimulatory effects on K^+^ transport systems in red cells from normal individuals and from other species, and may therefore mediate solute loss and red cell dehydration. The effect of XO and hypoxanthine (HO) on red cells, however, has not been detailed hitherto nor its effect on sickle cells. We postulated that oxidants produced by XO and HO may have deleterious effects on the permeability of sickle red cells and may therefore contribute to pathogenesis through solute loss. In this study, red cells from SCA patients were incubated with XO/HO mixtures, a well studied extracellular ROS‐generating system (Fridovich 1997; Baskurt et al. 1998; Wang et al. 1999). Levels of ROS within red cells were measured together with any effects on membrane transport pathways and red cell volume. Results show that, overall, oxidant generation elicited a reduction in sickling, Gardos channel, and P_sickle_ activities, whereas KCC activity and hemolysis in a nonelectrolyte assay were increased. An unexpected finding was maintenance of red cell volume in oxidant‐treated, deoxygenated sickle cells.

## Methods

### Reagents

Unless otherwise stated, reagents were purchased from Sigma Chemical Co. (Poole, Dorset, UK). Clotrimazole was purchased from Calbiochem (Nottingham, Notts., UK) and 5‐ (and‐6)‐chloromethyl‐2′,7′‐dichlorodihydro‐fluorescein diacetate (CM‐H_2_DCF‐DA) from Invitrogen Molecular Probes (Eugene, Oregon, USA). ^86^Rb^+^ was supplied by Perkin Elmer (Beaconsfield, Bucks., UK) and nitrogen by BOC Ltd (Guildford, Surrey, UK).

### Sample collection and handling

Blood samples were taken for routine tests according to clinical indications from patients with SCA (HbSS genotype) using the anticoagulant EDTA at King's College Hospital, London. After routine hematological testing, excess, anonymized blood samples were used for this study. All research was conducted with ethical approval and in accordance with the Helsinki Declaration of 1975, as revised in 2008.

### Solutions and tonometry

The standard saline (Cl‐MBS) which contained Ca^2+^ comprised (in mM) NaCl 145, CaCl_2_ 1.1, glucose 5, and 3‐(*N*‐morpholino)‐propane sulfonic acid (MOPS) 10, (pH 7.4 at 37^°^C; 290 ± 5 mOsm·kg^−1^ H_2_O). For experiments in which Cl^−^ dependence of K^+^ influx was examined, NO_3_^−^‐containing salts replaced those containing Cl^−^ (*N*‐MBS). For measurement of Gardos channel activity in Ca^2+^‐loaded red cells, high potassium (HK)‐containing MBS (HK‐MBS) was used comprising (in mmol/L) NaCl 70, KCl 80, and CaCl_2_ 0.01, together with MOPS (10 mmol/L) and glucose (5 mmol/L), to prevent rapid cell shrinkage following maximal activation of the channel. In these experiments, red cells were also exposed to the Ca^2+^ ionophore A23187 (6 *μ*mol/L). The wash solution to remove unincorporated radioisotope (^86^Rb^+^) comprised isotonic MgCl_2_ (107 mmol/L), buffered with MOPS (10 mmol/L), pH 7.4 at 4°C (Mg‐MBS). Stock solutions of bumetanide (10 mmol/L), ouabain (10 mmol/L), and clotrimazole (CLT; 5 mmol/L) were prepared in 100 mmol/L Tris base, distilled water, and dimethylsuldoxide, respectively. Whole blood was washed five times in *N*‐MBS to remove Cl^−^, plasma, and buffy coat. For most experiments, red cells (20% hematocrit, Hct) were then preincubated in air at 37°C for 15 min in the absence or presence of XO/HO mixtures, and the same XO/HO mixtures remained present throughout subsequent experimental manipulations. Red cell suspensions (still 20% Hct) in *N*‐MBS were then placed in tonometers (Eschweiler, Kiel, Germany) flushed with warm, humidified gas mixtures for 20 min at 37°C to equilibrate at the requisite O_2_ tension before measurements of K^+^ influx and red cell morphology (Speake et al. 1997). Gas mixtures were made using a Wösthoff gas mixing pump (Speake et al. 1997). Three oxygen tensions were used: 100 mmHg oxygen and 0 mmHg to oxygenate and deoxygenate red cells fully, and an intermediate tension of 30 mmHg at which HbS is about half saturated with oxygen, at which intracellular oxidant production is highest (Abugo and Rifkind 1994; Balagopalakrishna et al. 1996). For flux measurements, red cell suspensions were then diluted 10‐fold into flux tubes, still equilibrated at the same requisite O_2_ tension. To analyze red cell shape, aliquots of cells were fixed in saline containing 0.3% glutaraldehyde before examination under light microscopy counting typically around 300 cells (Hannemann et al. 2015).

### The reactive oxygen species‐generating system and measurement of intracellular ROS levels

Oxidants including hydrogen peroxide and superoxide anion were generated extracellularly by incubation of red cells with mixtures of HO and different activities of XO, a system which has previously been widely used to expose red cells to oxidative challenge (Baskurt et al. 1998; Rogers et al. 2009, 2013). For most experiments, red cells (20% Hct) were preincubated in air at 37°C for 15 min with this ROS‐generating system, and the same XO/HO mixtures remained present throughout subsequent experimental manipulations. Using data from control experiments, most work was carried out with a XO activity of 0.025 units·mL^−1^ with a HO concentration of 2 mmol/L. To measure oxidative load in RBCs, cells were loaded with CM‐H_2_DCF‐DA (10 *μ*mol/L) for 30 min at 37°C in the dark and then washed once in saline. On permeation into red cells, this fluorophore is deacetylated to the nonfluorescent di‐hydro compound which in the presence of ROS is oxidized to the highly fluorescent CM‐DCF. Fluorescence was measured in the FL‐1 channel of a BD Accuri™ C6 fluorescence‐activated flow cytometer (BDBiosciences, Oxford, UK) at an excitation wavelength of 488 nm and an emission wavelength of 519 nm. Measurements were taken using logarithmic gain. Forward scatter (FSC, size) and side scatter (SSC, granularity) gates for RBCs were identified in control experiments using anti‐glycophorin A‐PE‐labeled red cells (Cytlak et al. 2013). The positive fluorescent gate was set using red cells unexposed to CM‐H_2_DCF‐DA. For each measurement, 10,000 events were gated. CM‐DCF‐positive cells were defined as all events falling within the preset FSC, SSC, and positive fluorescent gates.

### K^+^ flux measurements

To determine the activity of the K^+^ transport pathways, K^+^ influx was measured using ^86^Rb^+^ as a congener for K^+^ (Dunham and Ellory 1981; Hannemann et al. 2011) at 37°C. Red cells were taken from tonometers and diluted 10‐fold into saline, pre‐equilibrated at the appropriate O_2_ tension at 260 mOsm·kg^−1^ (by addition of 10% water to the appropriate standard MBSs) and pH 7. ^86^Rb^+^ was added in 150 mmol/L KNO_3_ to give a final [K^+^] of 7.5 mmol/L. Three flux conditions were used in the flux tubes: (i) Cl‐MBS, (ii) Cl‐MBS with clotrimazole (5 *μ*mol/L), and (iii) *N*‐MBS with clotrimazole (5 *μ*mol/L). Ouabain (100 *μ*mol/L) and bumetanide (10 *μ*mol/L) were present in all experiments to obviate any K^+^ transport through the Na^+^/K^+^ pump and the Na^+^‐K^+^‐2Cl^−^ cotransporter, respectively. After incubation with radioisotope for 10 min, red cells were washed five times in ice‐cold Mg‐MBS wash solution to remove extracellular ^86^Rb^+^. Following the final wash, the cell pellet was lysed with Triton X‐100 (0.1%) and protein precipitated with trichloroacetic acid (5%). Activity was then measured as Čerenkov radiation by liquid scintillation (Packard Tri‐carb 2800TR, Perkin Elmer). P_sickle_ was assayed as the deoxygenation‐induced, CLT‐independent K^+^ influx measured in the absence of Cl^−^ (condition iii); Gardos channel activity as the CLT‐sensitive (5 *μ*mol/L) K^+^ influx (using conditions [i] and [ii]); and KCC activity was assayed as Cl^−^‐dependent K^+^ influx in the presence of CLT (using flux conditions [ii] and [iii]). As CLT and A23187 were dissolved in DMSO, controls were all treated with the same concentration of this solvent (0.1% final). Either the microhematocrit determination or the cyanohemoglobin method was used to measure the hematocrit (Hct) with appropriate samples taken at the start of each experiment (Speake et al. 1997; Hannemann et al. 2011).

### Nonelectrolyte hemolysis assay

Previous work has shown that hemolysis of sickle cells in deoxygenated nonelectrolyte solutions provides a simple measure of P_sickle_ activity (Browning et al. 2007; Milligan et al. 2013). The effect of XO/HO mixtures on deoxygention‐induced hemolysis in nonelectrolye solution was therefore tested. Washed red cells were suspended in isosmotic sucrose solution (290 mOsm·kg^−1^, pH 7.4 at 37°C), whose composition followed that of the standard MBS but in which all salts were replaced with sucrose (255 mmol/L), and deoxygenated in tonometers for 60 min. To measure hemolysis, aliquots of the suspension were taken every 10 min, intact red cells pelleted by centrifugation and the optical density (OD) of the supernatant measured at 540 nm. Values for 100% hemolysis were obtained from similar aliquots diluted into 0.1% Triton X‐100.

### Red cell volume determinations

The effect of XO/HO mixtures on the cell volume of deoxygenated sickle cells was investigated following incubation in the absence or presence of the oxidants for 60 min at pH 7.0 and 37°C, 5% Hct. Red cell water content was measured at the start and end of the incubation period using the wet weight – dry weight method of (Borgese et al. 1991). In brief, red cells were pelletted by centrifugation at 12,000*g* for 15 min at 4°C. The extruded pellet was weighed immediately (to an accuracy of 0.01 mg) and again after drying for 18 h at 95°C. Water content was expressed as ml water per g dry cell solids (mL·(g d.c.s.)^−1^). To measure trapped extracellular water, red cells were incubated as above but then quickly cooled in Eppendorf tubes in the presence of clotrimazole, ouabain, and bumetanide, by incubation in ice‐cold water for 10 min. Samples were divided into two aliquots and ^86^Rb^+^ was added to one. Both red cell aliquots, without or with ^86^Rb^+^, were layered over dibutyl phthalate oil (0.5 mL) whose density is such that on centrifugation (12,000 g, 10 sec) the oil allows the passage of cells only, while excluding any extracellular water which was not trapped between the red cells. The supernatant above the oil was aspirated and the tube interior surfaces carefully washed twice with ice‐cold Mg‐MBS. The oil layer was then also removed, leaving the red cell pellet together with trapped extracellular water. The red cell pellet from the aliquot to which ^86^Rb^+^ had been added was lysed with Triton X‐100 (0.1%) and the released protein was then precipitated with trichloroacetic acid (5%). Activity, representing trapped ^86^Rb^+^ in extracellular water, was then measured as Čerenkov radiation by liquid scintillation. The red cell pellet from the second aliquot was lysed in Drabkin's solution and its OD measured at 540 nm to find the total Hb present. The percentage of total ^86^Rb^+^ in the cell pellet, normalized to Hb content, was then compared in control red cells in the absence of oxidant and those treated with the XO/HO mixture.

### Statistics

**Figure 1:**
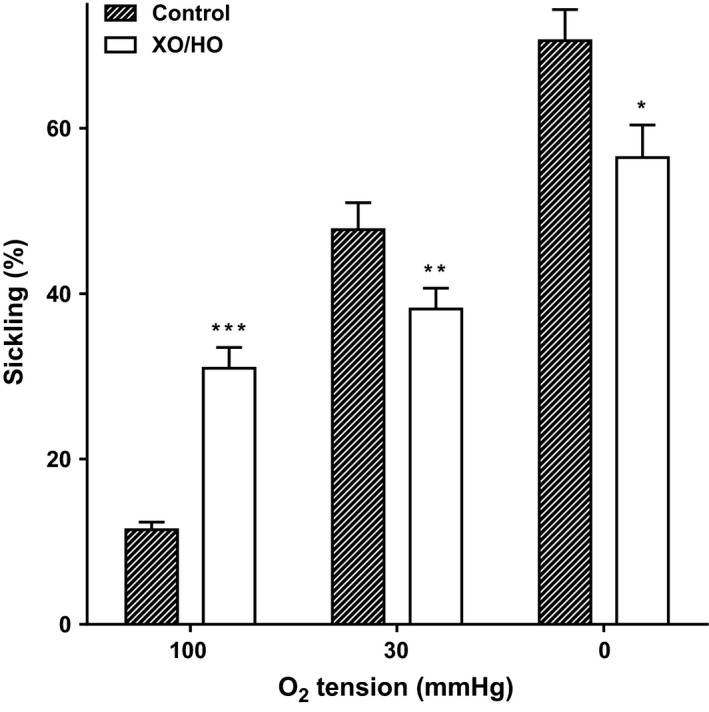
Effect of xanthine oxidase/hypoxanthine (XO/HO) mixtures on sickling of red cells from patients with sickle cell anemia (SCA). Red cells (20% hematocrit, Hct) from patients homozygous (HbSS) for SCA were preincubated at 37°C in air with or without XO (0.025 units·mL^−1^) and HO (2 mmol/L) in Cl‐MBS for 15 min. They were then equilibrated in Eschweiler tonometers for 20 min at the required oxygen tension (100, 30, and 0 mmHg oxygen) in the continued presence of the oxidant‐generating system or its absence, after which aliquots were fixed with glutaraldehyde (0.3%) and sickling was determined by light microscopy. Histograms represent means ± S.E.M., *n* = 9. * *P *<* *0.05, ** *P *<* *0.01, and *** *P *<* *0.001, comparing red cells in the absence and presence of XO/HO.

**Figure 2:**
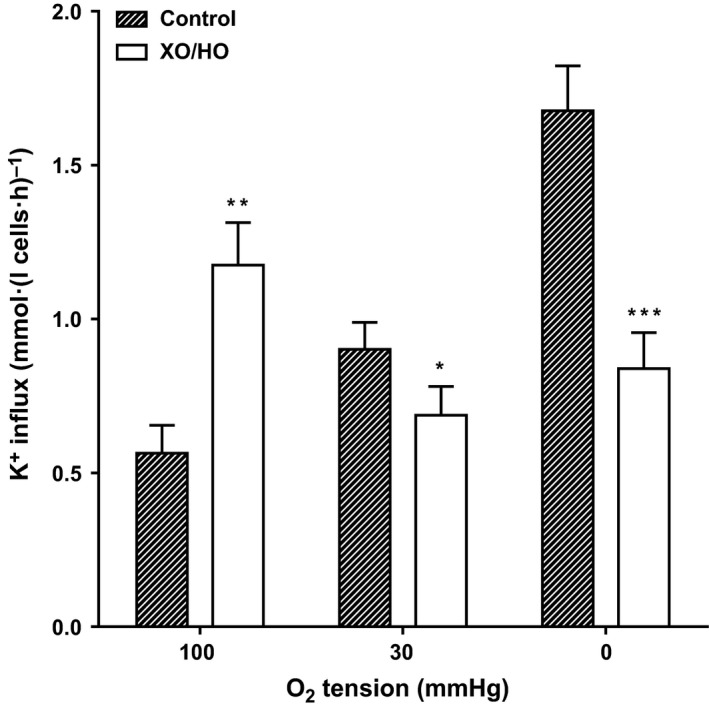
Effect of XO/HO mixtures on P_sickle_ activity in red cells from patients with SCA. Red cells from patients homozygous (HbSS) for SCA were preincubated with or without XO/HO in *N*‐MBS, as described in the legend to Figure 1. They were then equilibrated in Eschweiler tonometers for 20 min at the required oxygen tension, after which aliquots were diluted 10‐fold into flux tubes, all in the continued presence of the oxidant‐generating system or its absence. P_sickle_ activity, at an extracellular [K^+^] of 7.5 mmol/L and defined as the K^+^ influx in *N*‐MBS in the presence of clotrimazole (5 *μ*mol/L), was measured over 10 min and given in mmol K^+^.(L cells·h)^−1^. Ouabain (100 *μ*mol/L) and bumetanide (10 *μ*mol/L) were also present. Histograms represent means ± S.E.M., *n* = 9. *P <* *0.05, **P <* *0.01, and ***P <* *0.001, comparing red cells in the absence and presence of XO/HO.

Results are presented as means ± S.E.M. in red cell samples from n different patients. Those in Figures 1 (for sickling), 2 (for P_sickle_ activity), 3 (for Gardos channel activity), and 5 (for KCl cotransport activity) represent data simultaneously obtained from experiments on samples from the same nine different sickle cell patients. Comparisons were made using paired two‐tailed Student's *t*‐tests, as all data are from paired experiments with red cells under control conditions in the absence of XO/HO mixtures and those in their presence. The level of significance used was *P *<* *0.05. Although there was variability between patients, as observed previously, the direction of changes in the paired samples elicited by the presence of XO and HO mixtures was the same in all cases, increasing statistical significance. Correlation of P_sickle_ and Gardos channel activities was analyzed using Pearson's correlation (r). Plot lines in Figure 2 were fitted by nonlinear regression using Graphpad Prism (La Jolla, CA, USA).

## Results

### Effect of xanthine oxidase/hypoxanthine on intracellular ROS levels

Preliminary work was undertaken to establish levels of XO/ HO required to generate peak intracellular oxidant load in red cells (measured by CM‐DCF fluorescence). After 15 min, in the presence of HO (2 mmol/L), the intensity of red cell CM‐DCF fluorescence progressively increased as XO activity was raised, reaching a plateau at an activity of 0.025 units·mL^−1^ (50,788 ± 8081 arbitrary units) with no significant change as XO activity was further elevated to 0.05 or 0.10 units·mL^−1^ (50,978 ±6729 and 53,187 ± 9831 arbitrary units, respectively; both *P *=* *0.9 cf value at 0.025 units·mL^−1^). This combination of HO and XO activity (2 mmol/L and 0.025 units·mL^−1^, respectively) was selected for further study and represents a similar order of magnitude to those used previously (e.g Baskurt et al. 1998; Rogers et al. 2009).

### Effect of XO/HO mixtures on sickling, P_sickle_, and Gardos channel activities

**Figure 3:**
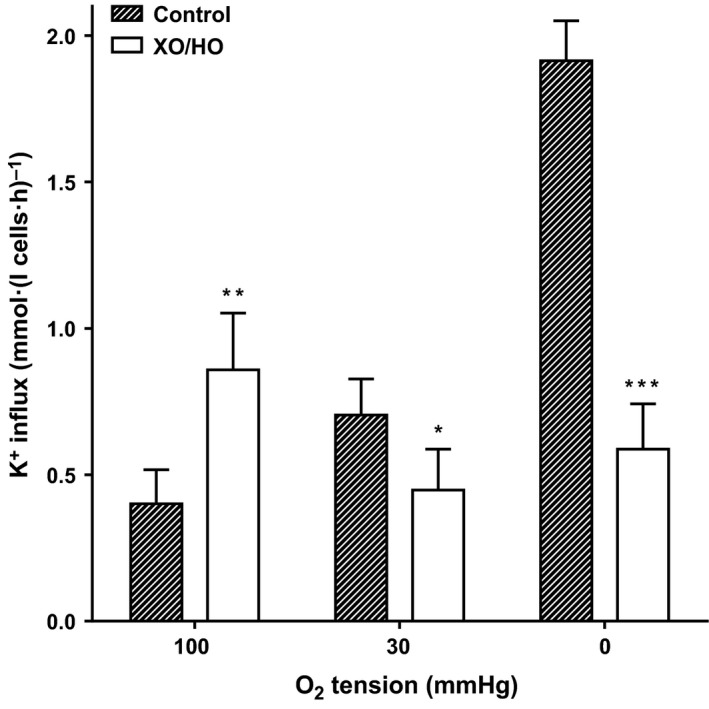
Effect of XO/HO mixtures on Gardos channel activity in red cells from patients with SCA. Red cells from patients homozygous (HbSS) for SCA were preincubated with or without XO/HO in N‐MBS, as described in the legend to Figure 1. They were then equilibrated in Eschweiler tonometers for 20 min at the required oxygen tension, after which aliquots were diluted 10‐fold into flux tubes, all in the continued presence of the oxidant‐generating system or its absence. Gardos channel activity, at an extracellular [K^+^] of 7.5 mmol/L and defined as the clotrimazole (5 *μ*mol/L)‐sensitive K^+^ influx in Cl‐MBS, was measured over 10 min and given in mmol K^+^.(L cells·h)^−1^. Ouabain (100 *μ*mol/L) and bumetanide (10 *μ*mol/L) were also present. Histograms represent means ± S.E.M., *n* = 9. *P <* *0.05, **P <* *0.01, and ***P <* *0.001, comparing red cells in the absence and presence of XO/HO.

**Figure 4:**
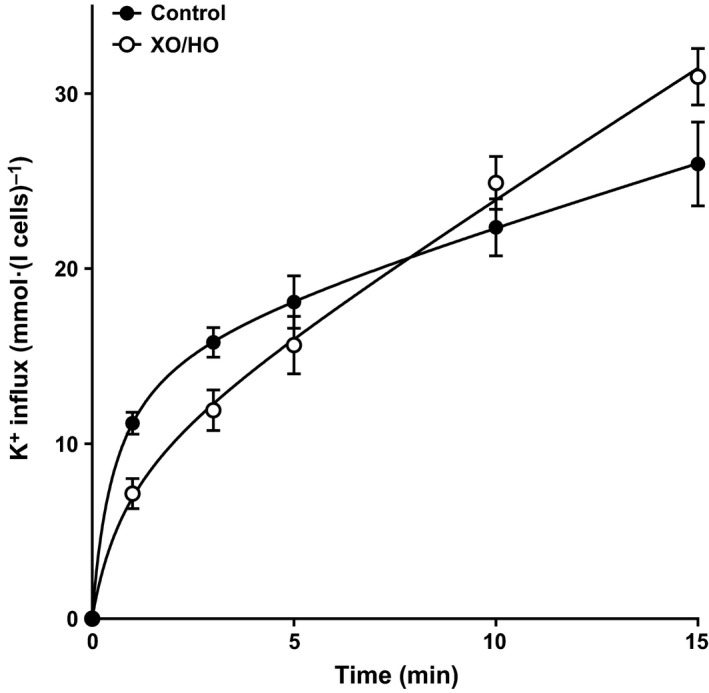
Effect of superoxide anion on Gardos channel activity in Ca^2+^‐loaded red cells from patients with SCA. Red cells from patients homozygous (HbSS) for SCA were preincubated with or without XO/HO, as described in the legend to Figure 1. They were suspended in air in HK‐MBS containing 10 *μ*mol/L CaCl_2_, and equilibrated in test tubes in the presence of the Ca^2+^ ionophore A23187 (6 *μ*mol/L) for 15 min, all in the continued presence of the oxidant‐generating system or its absence. Gardos channel activity, at an extracellular [K^+^] of 80 mmol/L and taken as the clotrimazole (5 *μ*mol/L)‐sensitive K^+^ influx, was measured at various time points over 15 min and given in mmol K^+^.(L cells)^−1^. Ouabain (100 *μ*mol/L) and bumetanide (10 *μ*mol/L) were also present. Symbols represent means ± S.E.M., *n* = 5. The curves are fitted by nonlinear regression using Graphpad Prism software which gave an apparent *V*_max_ of 27.5 ± 1.8 mmol K^+^.(L cells)^−1^ in the absence of XO/HO and 50.4 ± 6.1 mmol K^+^.(L cells)^−1^ in its presence (*P *<* *0.0001), and a half‐time of 2.0 ± 0.48 min and 9.9 ± 2.3 min (*P *<* *0.0001), respectively.

In the first set of experiments, the effect of XO/HO mixtures was examined on red cell morphology (Fig. 1). In fully oxygenated red cells, sickling was increased in the presence of XO/HO mixtures while at intermediate oxygen tensions and in fully deoxygenated red cells sickling levels were reduced. These effects were significant at all three oxygen tensions. A similar pattern was observed for P_sickle_ activity (Fig. 2). The effect of XO/HO mixtures on Gardos channel activity also showed the same relationship with that of sickling and P_sickle_ – increase in fully oxygenated cells (Fig. 3; *P *<* *0.01) and decrease at intermediate oxygen tensions (*P *<* *0.05) and full deoxygenation (*P *<* *0.001). In deoxygenated red cells, P_sickle_ and Gardos channel activities correlated significantly in the absence of XO/HO mixtures (*r* = 0.81; *P *<* *0.02) but not in their presence (*P *=* *0.29). Similarly, combining channel activities from all three oxygen tensions, significant correlation was present in control red cells (*r* = 0.78; *P *<* *0.0001) but not in cells treated with oxidants (*r* = 0.01; *P *=* *0.94). With red cells loaded with Ca^2+^ using the ionophore A23187 (6 *μ*mol/L) in MBS containing 10 *μ*mol/L CaCl_2_ to activate the Gardos channel pharmacologically, the rate of K^+^ influx (V_max_) and half‐time (t_1/2_) were significantly increased in the presence of XO/HO mixtures (Fig. 4). These findings probably indicate an action of oxidants on the Gardos channel per se, as well as through its effects on Ca^2+^ entry via P_sickle_ activity (as in Fig. 2).

### Effect of XO/HO mixtures on KCl cotransport activity

**Figure 5:**
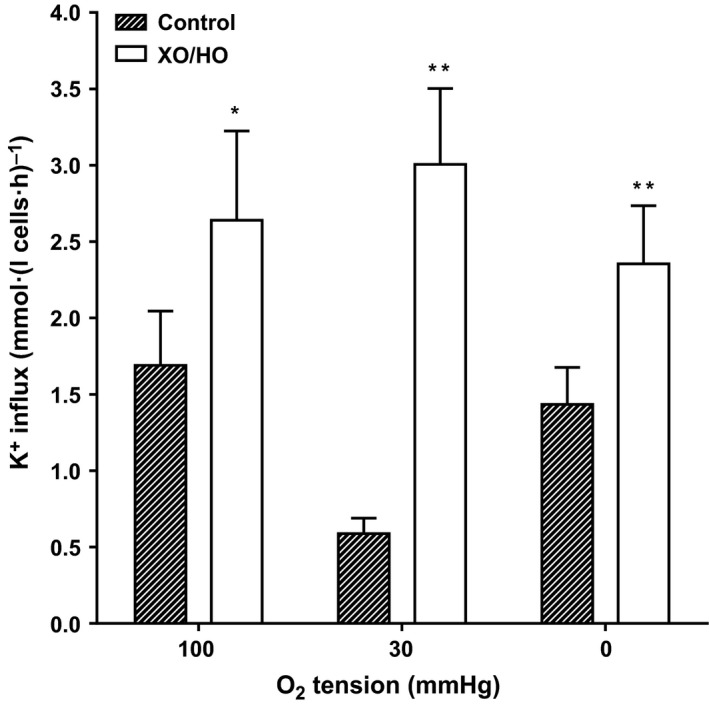
Effect of XO/HO mixtures on KCl cotransport activity in red cells patients with SCA. Red cells from patients homozygous (HbSS) for SCA were preincubated with or without XO/HO in *N*‐MBS, as described in the legend to Figure 1. They were then equilibrated in Eschweiler tonometers for 20 min at the required oxygen tension, after which aliquots were diluted 10‐fold into flux tubes, all in the continued presence of the oxidant‐generating system or its absence. KCl cotransport (KCC) activity, at an extracellular [K^+^] of 7.5 mM and defined as the K^+^ influx in the presence and absence of Cl^−^, was measured over 10 min and given in mmol K^+^.(L cells·h)^−1^. Ouabain (100 *μ*mol/L), bumetanide (10 *μ*mol/L), and clotrimazole (5 *μ*mol/L) were also present. Histograms represent means ± S.E.M., *n* = 9. * *P *<* *0.05 and ** *P *<* *0.01, comparing red cells in the absence and presence of XO/HO.

**Figure 6:**
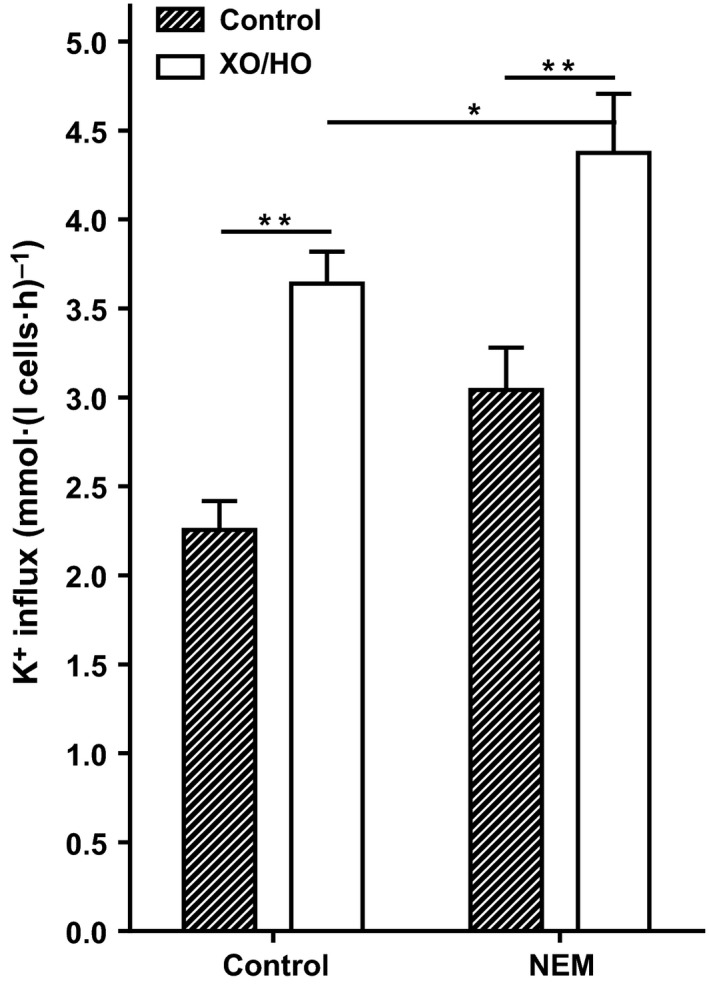
Effect of *N*‐ethylmaleimide on KCl cotransport activity in red cells patients with SCA. Red cells from patients homozygous (HbSS) for SCA were first preincubated in *N*‐MBS without or with *N*‐ethylmaleimide (NEM, 1 mmol/L) for 45 min at 37°C, 20% Hct. Both aliquots were then divided and subsequently incubated in air with or without the oxidant‐generating system, as described in the legend to Figure 1, after which aliquots were diluted 10‐fold into flux tubes, all in the continued presence of the oxidant‐generating system or its absence. KCl cotransport (KCC) activity, at an extracellular [K^+^] of 7.5 mM and defined as in the legend to Figure 5, was measured over 10 min and given in mmol K^+^.(L cells·h)^−1^. Ouabain (100 *μ*mol/L), bumetanide (10 *μ*mol/L), and clotrimazole (5 *μ*mol/L) were also present. Histograms represent means ± S.E.M., *n* = 6. *P <* *0.05 and **P <* *0.01.

The third main cation transport pathway involved in sickle cell dehydration is the KCl cotransporter (KCC). KCC activity was measured at the three oxygen tensions used to examine the behavior of sickling, P_sickle_ and Gardos channel. At all oxygen tensions, KCC activity was significantly elevated in the presence of XO/HO mixtures, with the largest increment observed at intermediate oxygen tension (Fig. 5; *P *<* *0.05, 0.01 & 0.01 at 100, 30 & 0 mmHg oxygen, respectively). Effects on KCC activity may be mediated directly on the transporter or on the regulatory kinase and phosphatase cascade which modulates activity. This was examined using *N*‐ethylmaleimide (NEM, 1 mmol/L)‐treated red cells. NEM is a sulfydryl reacting reagent is thought to act via inhibition of red cell kinase activity resulting in full activation of the KCl cotransporter (Ellory et al. 1982; Lauf 1984) with loss of regulation by phosphoresidues. As expected, NEM treatment (1 mmol/L for 30 min at 37°C, 20% Hct) resulted in raised KCC activity (Fig. 6). XO/HO mixtures, however, were still observed to significantly elevate KCC activity in both control (absence of NEM) and NEM‐treated red cells (both *P *<* *0.01), suggesting that at least part of its action must be directly on the transporter as opposed to regulatory enzymes.

### Effect of XO/HO mixtures on nonelectrolyte hemolysis

**Figure 7:**
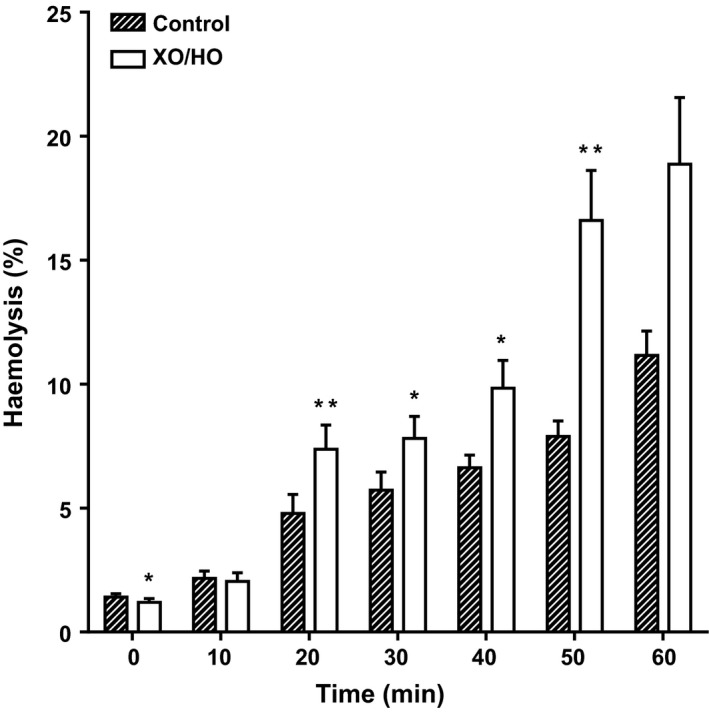
Effect of XO/HO mixtures on nonelectrolyte‐induced hemolysis of red cells patients with SCA. Red cells from patients homozygous (HbSS) for SCA were preincubated with or without XO/HO in Ca^2+^‐free *N*‐MBS, as described in the legend to Figure 1. They were then incubated in isosmotic nonelectrolyte solution in Eschweiler tonometers and equilibrated with nitrogen for 60 min. Hemolysis (% total) was measured at 10 min intervals by removing serial aliquots of red cell suspensions, pelletting intact red cells by centrifugation, and measuring the optical density (O.D.) of the supernatant at 540 nm. A 100% hemolysis was ascertained by measuring O.D. in red cell suspensions treated with X‐100 Triton (0.1% final). Histograms represent means ± S.E.M., *n* = 4. * *P* < 0.05 and ** *P* < 0.01 comparing red cells in the absence and presence of XO/HO.

Previous work has shown that hemolysis of sickle cells in deoxygenated nonelectrolyte solutions provides a simple measure of P_sickle_ activity. The effect of incubation in XO/HO mixtures was tested therefore here. Results showed that hemolysis was increased in the presence of oxidants, becoming significant after 20 min (Fig. 7; *P *<* *0.01). By 60 min incubation, hemolysis with oxidants was almost double that observed in control samples.

### Effect of XO/HO mixtures on red cell volume

**Figure 8:**
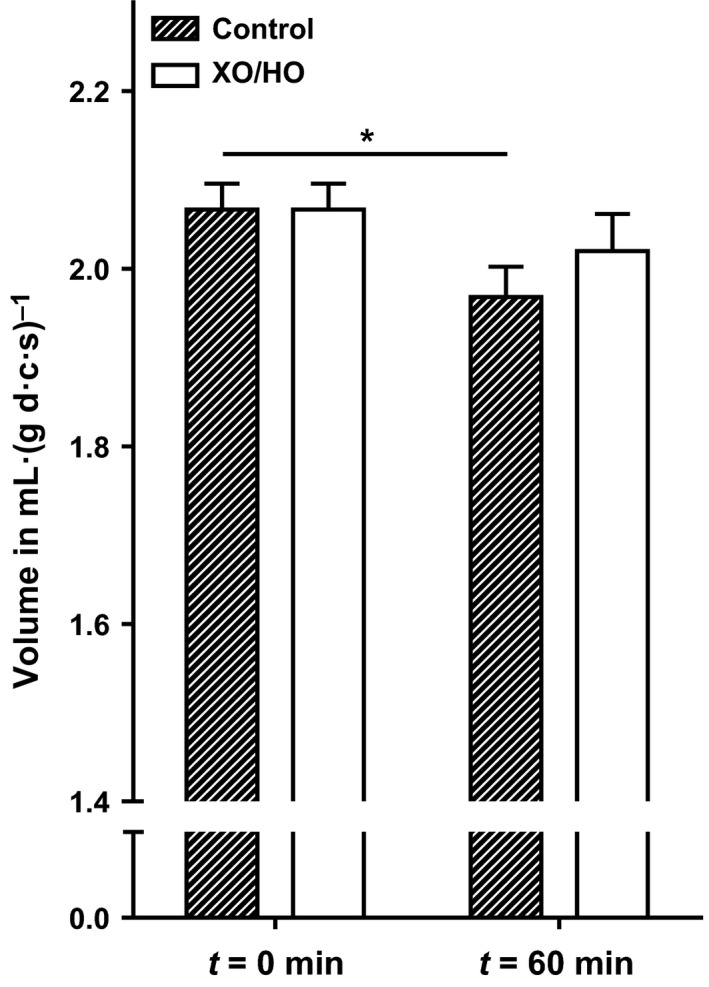
Effect of XO/HO mixtures on cell water content of red cells patients with SCA. Red cells from patients homozygous (HbSS) for SCA were preincubated with or without XO/HO, as described in the legend to Figure 1. They were then incubated for 60 min at 0 mmHg oxygen tension, after which cell water content, in ml.(g d.c.s.)^−1^, was measured using the method of Borgese et al. 1991, all in the continued presence of the oxidant‐generating system or its absence. Histograms represent means ± S.E.M., *n* = 6. *P < 0.05 comparing red cell water content after 60 min incubation to that at *t* = 0, in cells incubated in the absence of XO/HO (control).

Finally, the effect of XO/HO mixtures was investigated on red cell water content. Red cell aliquots were incubated under deoxygenated conditions at pH 7 for 60 min in the absence or presence of XO/HO mixtures. In the absence of oxidants, there was a significant reduction in red cell volume (Fig. 8; *P *<* *0.05). In the presence of XO/HO mixtures, however, red cell volume was maintained at magnitudes not significantly different from those observed at the start of the experiment (*t* = 0 min), although a modest reduction did occur. Cell volume in the presence of XO/HO mixtures was also greater in deoxygenated red cells compared to those in the absence of oxidants, although this effect was not significant. Trapped extracellular water due to a difference in red cell deformability between control and XO/HO mixture treated red cells may affect the measured red cell water content. Although trapped extracellular water was slightly reduced in the presence of XO/HO mixtures (92.7 ± 10%, *n* = 4, cf control cells), this effect was not significant (*P *=* *0.5).

## Discussion

The effects of XO/HO mixtures on phenotype were investigated in red cells from patients with SCA. Results showed that sickling, P_sickle_, and Gardos channel activities were increased in the presence of oxidants when cells were fully oxygenated, but were reduced at the other oxygen tensions. In comparison, KCC activity was increased by XO/HO mixtures at all three oxygen tensions. The level of deoxygenation‐induced nonelectrolyte hemolysis was also elevated. Under the conditions tested, however, the overall effect of this oxidant‐generating system was to stabilize red cell volume and inhibit dehydration.

Red cells from patients with SCA are exposed to greater oxidant challenge compared with those from normal individuals (Voskou et al. 2015). In addition, the antioxidant capacity of sickle red cells is lower than normal, although whether superoxide dismutase levels are reduced (Schacter et al. 1985, 1988; Ren et al. 2008), or even increased (Titus et al. 2004), is less clear, and may differ between individuals. Notwithstanding, exposure of sickle cells to oxidants is increased. XO/HO mixtures will generate both hydrogen peroxide and superoxide anion. While, in some respects, the superoxide anion is less reactive than many other ROS, it is a precursor of more aggressive ones, including hydrogen peroxide (following interaction with red cell superoxide dismutase) and the hydroxyl radical (following the Haber–Weiss and Fenton reactions) (Cheeseman and Slater 1993; Balagopalakrishna et al. 1996; Cimen 2008). Oxidants are also capable of directly attacking the red cell membrane causing lipid peroxidation and protein damage (Claster et al. 1984). These factors present a clear oxidative challenge to the sickle red cell as a whole, while its membrane may be particularly vulnerable (Mohanty et al. 2013) which is of especial relevance to systems regulating red cell cation permeability, solute content and volume.

Damaging effects of oxidants have previously been demonstrated on both the cytoplasmic cytoskeleton and also the protein and lipid components of the membrane of red cells (Baskurt et al. 1998). A reduction in red cell deformability has been reported (Hebbel et al. 1990; Watanabe et al. 1990; Uyesaka et al. 1992; de Jong et al. 1997; Baskurt et al. 1998; Barodka et al. 2014; Kuypers and de Jong 2014). Such an effect may reduce sickling and hence P_sickle_ activity, and also P_sickle_‐mediated Ca^2+^ entry. Lower levels of sickling, P_sickle_, and Gardos channel activities in deoxygenated sickle cells observed in the present work are consistent with these actions. Interestingly, however, although P_sickle_ and Gardos channel activities correlated in deoxygenated control cells, consistent with Gardos channel activation subsequent to Ca^2+^ entry via P_sickle_, they did not following incubation with oxidants. It is possible that other membrane effects of oxidant challenge alters the permeability of the P_sickle_ pathway so that univalent and divalent cation permeabilities no longer correlate, consequently uncoupling P_sickle_ and Gardos channel activities. Conversely, inhibition of transport activities to lower values in the presence of oxidants may reduce the signal‐to‐noise ratio obscuring any correlation. In addition, the effect of oxidant challenge on the rate of K^+^ flux following pharmacological elevation of Ca^2+^ with the ionophore A23187, increasing both V_max_ and t_1/2_, suggests that there is also a significant action on the Gardos channel *per se*.

KCC activity was higher at all oxygen tensions tested. This was particularly the case at intermediate values (30 mmHg oxygen) where HbS would be about half saturated with oxygen and which is associated with maximal production of ROS (Abugo and Rifkind 1994; Balagopalakrishna et al. 1996; Mohanty et al. 2014). KCC is controlled by regulatory kinases and phosphatases and part of the action of oxidants may be mediated via these enzymes. Red cell oxidant damage, including that by hydrogen peroxide and superoxide anion, reduces levels of glutathione (GSH) (Rogers et al. 2009) which have been associated with increased activation of KCC activity probably acting via these enzymes (Lauf et al. 1995; Gibson and Muzyamba 2003a,b).

KCC activity was also elevated by XO/HO mixtures in red cells pretreated with NEM. This thiol alkylating agent is thought to activate KCC activity by abrogating the activity of the inhibitory protein kinase (Lauf 1983, 1984) and the significant increase in KCC activity in NEM‐treated red cells therefore probably indicates a direct effect on the transporter. In this context, superoxide anion itself has been previously shown to oxidize membrane thiols (Wang et al. 1999) while other oxidants including 1‐chloro‐2,4‐dinitrobenzene, phenazine methosulfate, nitrite and peroxynitrite have also been shown to increase red cell KCC activity (Muzyamba et al. 2000; Gibson and Muzyamba 2003a,b; Kucherenko et al. 2005).

As well as reducing glutathione levels, oxidants will cause formation of considerable methemoglobin (metHb) (Baskurt et al. 1998; Mohanty et al. 2013). MetHb is in the oxy conformation and has previously been associated with loss of inhibition of KCC at lower oxygen tensions (Muzyamba et al. 2000; Gibson and Muzyamba 2003a,b; Kucherenko et al. 2005). The oxy conformation of metHb would also be expected to reduce HbS polymerization, and hence sickling, and also sickling‐induced permeability changes, as is the case observed here. It is important to note, however, that bulk cytoplasmic Hb is probably less important for controlling membrane permeability than membrane‐associated Hb, as oxygen‐dependent KCC activity persists in pink ghosts from normal individuals (Drew et al. 2004) and a deoxygenation‐induced cation conductance with similar characteristics to P_sickle_ is also observed electrophysiologically in sickle cells in the whole‐cell patch clamp configuration (Ma et al. 2012). In both preparations, most cytoplasmic Hb will have been lost, but not that bound to the membrane. There is considerable evidence that it is the membrane‐bound component of total red cell Hb, which constitutes the molecular switch and is the key to oxygen‐sensitive properties of the red cells (Chu et al. 2016).

The effect of oxidants on hemolysis in deoxygenated nonelectrolyte solutions was also tested. This assay provides a simple measure of P_sickle_ activity (Browning et al. 2007; Milligan et al. 2013), and in many cases, nonelectrolyte hemolysis associates with P_sickle_ activity when measured as a cation flux. In the experiments described here, however, although K^+^ influx through P_sickle_ was inhibited by XO/HO mixtures, hemolysis was substantially increased. A similar effect was observed previously although at much higher levels of XO activity (0.16 units·mL^−1^; Gbotosho 2015). Oxidant‐mediated damage to components of the cytoskeleton notably spectrin (Schwartz et al. 1987; Shinar et al. 1989; Advani et al. 1992; Baskurt et al. 1998; Goodman 2004), and to membrane lipids and proteins may act to destabilize red cell integrity, thus uncoupling the activities of P_sickle_ cation fluxes and the extent of nonelectrolyte hemolysis.

Notwithstanding the effects of oxidants on several K^+^ transport systems in the membrane of sickle cells, measurement of red cell volume showed that while in the absence of oxidants deoxygenated red cells shrank over the course of an hour, in its presence shrinkage was reduced. The largely antagonistic effects of oxidant exposure on the activities of Gardos channel and KCC may cancel out and result in maintenance of cation content and cell volume. There are previous reports that oxidants increase the cell volume of red cells from normal individuals (Uyesaka et al. 1992). Notwithstanding, the effect of XO/HO mixtures on red cell water content was not due to differences in trapped extracellular water.

In conclusion, the effect of XO/HO mixtures on the permeability of sickle cells showed inhibitory actions on sickling, P_sickle_, and Gardos channel activities, while, by contrast, KCC activity and nonelectrolyte hemolysis were increased. Overall, red cell volume was maintained in oxygenated, deoxygenated and partially oxygenated red cells. The most notable effect of superoxide anion was thus probably reduction in red cell stability. These findings increase our understanding of the effects of oxidative challenge and are relevant to the behavior of red cells in vivo in SCA patients.

## Conflicts of Interest

There are no conflicts of interest.
